# An 8-week freeze-dried blueberry supplement impacts immune-related pathways: a randomized, double-blind placebo-controlled trial

**DOI:** 10.1186/s12263-021-00688-2

**Published:** 2021-05-17

**Authors:** Michèle Rousseau, Justine Horne, Frédéric Guénard, Juan de Toro-Martín, Véronique Garneau, Valérie Guay, Michèle Kearney, Geneviève Pilon, Denis Roy, Patrick Couture, Charles Couillard, André Marette, Marie-Claude Vohl

**Affiliations:** 1 Centre Nutrition, santé et société (NUTRISS)-Institut sur la nutrition et les aliments fonctionnels (INAF), Université Laval, Québec, QC G1V 0A6 Canada; 2 School of Nutrition, Université Laval, Québec, QC G1V 0A6 Canada; 3 Québec Heart and Lung Institute (IUCPQ) Research Center, 2725 chemin Sainte-Foy, Québec, QC G1V 4G5 Canada

**Keywords:** Metabolic syndrome, Blueberry, Overweight/obesity, Nutrition, Immunity, Transcriptomics, Metabolomics, Gene expression

## Abstract

**Background:**

Blueberries contain high levels of polyphenolic compounds with high *in vitro* antioxidant capacities. Their consumption has been associated with improved vascular and metabolic health.

**Purpose:**

The objective was to examine the effects of blueberry supplement consumption on metabolic syndrome (MetS) parameters and potential underlying mechanisms of action.

**Methods:**

A randomized double-blind placebo-controlled intervention trial was conducted in adults at risk of developing MetS. Participants consumed 50 g daily of either a freeze-dried highbush blueberry powder (BBP) or a placebo powder for 8 weeks (*n* = 49). MetS phenotypes were assessed at weeks 0, 4 and 8. Fasting blood gene expression profiles and plasma metabolomic profiles were examined at baseline and week 8 to assess metabolic changes occurring in response to the BBP. A per-protocol analysis was used.

**Results:**

A significant treatment effect was observed for plasma triglyceride levels that was no longer significant after further adjustments for age, sex, BMI and baseline values. In addition, the treatment*time interactions were non-significant therefore suggesting that compared with the placebo, BBP had no statistically significant effect on body weight, blood pressure, fasting plasma lipid, insulin and glucose levels, insulin resistance (or sensitivity) or glycated hemoglobin concentrations. There were significant changes in the expression of 49 genes and in the abundance of 35 metabolites following BBP consumption. Differentially regulated genes were clustered in immune-related pathways.

**Conclusion:**

An 8-week BBP intervention did not significantly improve traditional markers of cardiometabolic health in adults at risk of developing MetS. However, changes in gene expression and metabolite abundance suggest that clinically significant cardiometabolic changes could take longer than 8 weeks to present and/or could result from whole blueberry consumption or a higher dosage. BBP may also have an effect on factors such as immunity even within a shorter 8-week timeframe.

**Clinical trial registration:**

clinicaltrials.gov, NCT03266055, 2017

**Supplementary Information:**

The online version contains supplementary material available at 10.1186/s12263-021-00688-2.

## Introduction

With an estimated prevalence of almost 35% in the USA [1] and 19% in Canada [2], metabolic syndrome (MetS) is a condition of great concern in developed countries. By clustering abdominal obesity, insulin resistance (IR), dyslipidemia and hypertension, people with MetS are at higher risk of developing type 2 diabetes and cardiovascular disease [3]. Oxidative stress is a commonality for these risk factors and is suspected of playing a pro-inflammatory role thus promoting their development [4]. Oxidative stress occurs when the body’s antioxidant defenses are not sufficient to counterbalance production of free radicals and other reactive oxygen species (ROS) [4]. Free radicals are highly reactive and have the potential to damage essential biomolecules including lipids, proteins and DNA, which in turn impair their functions [5]. Notably, excess calorie intake leads to increased ROS production, which could eventually generate a pro-oxidant environment susceptible to cause cellular dysfunction promoting obesity-related diseases [4].

Nutrition has the potential to help mitigate issues related to increased ROS production. For example, many fruits are naturally rich in polyphenolic molecules with potent antioxidant and anti-inflammatory activity, which are part of their natural defense system against pathogens, environmental fluctuations and pro-oxidant stressors such as ultraviolet light [6]. Berries are among fruits with the highest polyphenol content, mainly anthocyanins, resulting in their bright color [7–9]. Compared with 25 other commonly eaten fruits, wild and cultivated blueberries were previously ranked first and fifth, respectively, for their total phenolic content (respectively 429 and 285 gallic acid equivalent per 100 g of fruit) [9]. Analyses of the effect of lowbush blueberry plant extracts on cultured cells also revealed insulin- and glitazone-like activities, suggesting that blueberries may possess antidiabetic properties [8, 10]. Blueberry supplementation has previously been associated with favorable effects on blood pressure [11, 12], some aspects of brain cognitive function [13, 14] and plasma biomarkers of oxidative stress and inflammation [11]. Blueberry consumption has also been demonstrated to be protective against the risk of myocardial infarction [15] and diabetes [16]. However, these results are inconsistent in the literature, with some clinical trials showing no improvement on lipid profile [11, 17–19], insulin resistance [11, 18, 20] or blood pressure [17–20]. Differences in study designs and populations tested [21] could explain these equivocal results, highlighting the need for further research on blueberry health properties. In addition, inter-individual variability in cardiometabolic responses to nutrition intervention is observed and can be partly attributable to genetic variations [22]. In order to inform future investigations specific to blueberries and polyphenols as they relate to MetS, a better understanding of the physiological mechanism of action for blueberries (polyphenols) on MetS phenotypes is needed.

Accordingly, this 8-week parallel, randomized (1:1) double-blind placebo-controlled clinical trial of adults at risk of MetS had two overarching purposes. First, the study aimed to evaluate the impact of highbush blueberry powder (BBP) on the following MetS parameters: plasma insulin resistance (HOMA-IR) and sensitivity (Matsuda index), plasma lipids, arterial blood pressure, blood glucose, weight, body mass index (BMI) and waist circumference. Second, the study aimed to delineate the mechanism of action of highbush BBP on MetS parameters in overweight/obese adults through analyses of changes in key molecular signalling pathways and metabolic regulatory networks identified using analyses of transcriptomics and metabolomics. It was hypothesized that BBP have a significant effect on cardiometabolic MetS phenotypes and pathways over an 8-week follow-up period.

## Methods

### Study population

Recruitment of study participants took place between September 2017 and November 2018 in the greater Québec City metropolitan area, with follow-up occurring between October 10, 2017 and January 14, 2019. All recruitment and intervention visits took place at the Institute of Nutrition and Functional Foods (INAF), Université Laval. Recruitment ended when the sample size was achieved. A sample size calculation using published data [17] indicated that a total of 28 subjects per group were needed to observe significant changes in fasting plasma triglyceride (TG) and insulin taking into account an anticipated dropout rate of 20%, with a power of 80%. Using group electronic messages sent to university and INAF members as well as newspapers and social media advertisements, a total of 657 individuals contacted the researchers for information on this study. Overall, 110 individuals met email or phone pre-screening criteria and were scheduled for a screening visit at the clinical investigation unit of INAF. Before each visit, participants were asked to follow a 12-h overnight fast and abstain from alcohol for 48 h. They were also asked to avoid vigorous exercise for 24 h prior the visit. Inclusion criteria were as follows: Caucasian men or premenopausal, non-pregnant and non-lactating Caucasian women in good health, having a BMI between 25 and 40 kg/m^2^ or a waist circumference ≥ 94 cm for men and ≥ 80 cm for women. All participants had to have at least one of the following: fasting plasma TG ≥ 1.35 mmol/L or fasting insulin concentration ≥ 42 pmol/L using our new analytic method and corresponding to a threshold value of 60 pmol/L with the former method that was predictive of a higher risk of cardiovascular disease in the Quebec population [23]. Participants were excluded from the study if they self-reported that they were diagnosed with diabetes, hypercholesterolemia or hypertension or that they were taking medications for these conditions; were allergic or intolerant to blueberries; had a taste aversion to blueberries; were taking medication affecting study parameters; had taken antibiotics, supplements or natural health products on a regular basis over the past 3 months, had undergone surgery in the last 3 months or had planned surgery during the duration of the study; were nicotine users; followed unique dietary patterns (such as a vegan, gluten-free or ketogenic diet); had lost or gained > 5% of their body weight in the last 3 months; or were having more than 2 alcoholic drinks per day on a regular basis. Participants also had to be willing to commit to the study instructions from the run-in period until the end of the intervention. These instructions were to eat a maximum of: 2 portions of berries weekly (including whole fruits, juice, jam and desert but excluding the placebo/blueberry powders); 140 g of food containing cocoa weekly; 1 cup of tea or 4 cups of coffee daily; and 2 alcoholic drinks per week. Red wine and port were prohibited due to their flavonoid content. Participants were also asked to report the use of new medications and to avoid natural health product consumption or changes in lifestyle habits. The inclusion/exclusion criteria were adjusted following the commencement of the trial to enhance recruitment (original criteria: BMI 25.0–40.0 kg/m^2^ *and* abdominal obesity; insulin ≥ 60 pmol/L *and* TG ≥ 1.5 mmol/L); the criteria listed above reflect the revised, most current criteria. There were no other changes to the methods or outcomes after the trial commenced. The inclusion criteria can be found on the clinical trial registry at https://clinicaltrials.gov/ (NCT03266055). This study was approved by the Ethics Committee of Université Laval. All subjects signed a written informed consent prior their participation to the study.

### Study protocol and intervention

This study is a randomized double-blind placebo-controlled clinical intervention. Sex-stratified block randomization was applied electronically on the INAF electronic management platform to ensure a 1:1 treatment ratio for each sex to either blueberry (BBP) or placebo powder. Powders were both provided by the US Highbush Blueberry Council. The BBP consisted of a blend of milled freeze-dried highbush blueberries from two cultivars (*Vaccinium virgatum* (ashei) and *Vaccinium corymbosum*), in a 1:1 ratio. The placebo powder was isocaloric and of similar aspect and taste as the BBP, but contained a mixture of dextrose, maltodextrin, fructose, citric acid, malic acid, natural and artificial flavor, xanthan gum, silicon dioxide, and FD & C Red 40 and Blue 2 lakes. Both powders were packaged in identical aluminum packets identified with the letters A or B (for blinding) by the US Highbush Blueberry Council before sending packets to the study centre. Sufficient powder packets were provided at each visit to participants. They had to take a total of 50 g of powder daily divided in two packets of 25 g each, taken at least 8 h apart. Fifty grams of freeze-dried BBP is equivalent to about 350 g (2 and 1/3 cups) of fresh blueberries. Participants were asked to dilute the content of each packet in 300 ml water in a plastic cup provided by the study coordinator or to mix it into foods that were already part of their usual diet such as breakfast cereals, milk, yogurt or smoothies. They were asked not to heat the powder or add it to a hot liquid or food. To assess possible adverse events, participants completed a self-administered side effects questionnaire at weeks 4 and 8. Changes in plasma insulin, glucose, lipids and lipoproteins were primary outcomes. Changes in gene expression, metabolites, and blood pressure were secondary outcomes. All other analyses were exploratory.

**Fig. 1:**
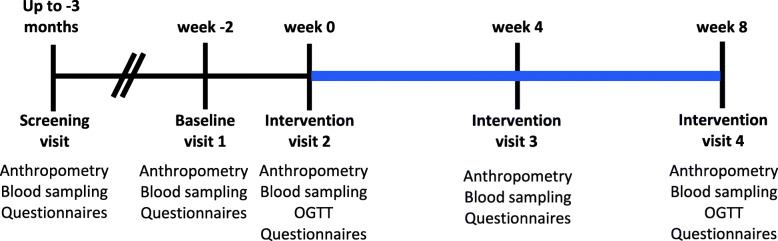
Graphical representation of the study protocol. Study design graphical representation from recruitment to the end of the supplementation period. The blue line represents the intervention period. Abbreviation: *OGTT*, oral glucose tolerance test

When eligibility was confirmed, participants were enrolled in the intervention and invited to the research institute for the baseline visit (week − 2) prior to a 2-week run-in period (Fig. 1). In that period, participants had to commit to the abovementioned study directives without taking the powder. At the start of the intervention (week 0), participants were randomized to consume either the BBP or the placebo powder for 8 weeks and scheduled for visits at the research center at 4-week intervals (weeks 4 and 8). The randomization list was created by VGa. Clinical coordinators (VGa, MK, VGu) were responsible for enrolling participants and assigning them to interventions. Compliance to the study protocol and supplementation was documented by the completion of a journal and by returning all powder packets (both empty and remaining) at each visit.

### Dietary assessment and questionnaires

Dietary habits were assessed on weeks 0, 4 and 8 by a web-based, self-administered, past-month food frequency questionnaire (FFQ). This FFQ was previously validated for French-speaking Canadian adults [24]. Briefly, participants were asked to report their consumption of 136 items grouped in 8 categories including dairy products, fruits, vegetables, meat and alternatives, cereals and grain products, beverages, “other foods” and supplements. Powders’ nutritional values were added to intakes reported on the FFQs completed at week 4 and week 8 with consideration of individual compliance percentage. Women and men with intakes lower than 600 or 800 kcal per day or greater than 3500 or 4200 kcal/day, respectively, were considered as under- or over-reporters and as such, their dietary intakes were not included in the analyses [25]. On the final visit, all participants completed a short questionnaire about the powders’ taste acceptability and consumption.

### Anthropometric measures

Weight was measured using a BWB-800 electronic scale (Tanita, Arlington Heights, IL) to the nearest 0.1 kg with patients wearing light indoor clothes and no shoes. Height and waist circumference were measured to the nearest millimeter according to procedures recommended at the Airlie conference [26]: waist was defined as the midpoint between the last floating rib and the top of the iliac crest. The mean of three consecutive measures was used for analyses. Systolic (SBP) and diastolic (DBP) blood pressure were measured while sitting on a chair after a 10-min rest. Again, the mean of three measures repeated at 3-min intervals was used for analyses.

### MetS phenotype parameters

Blood samples were drawn at each visit. Fasting plasma lipid profile (total cholesterol (Total-C), HDL cholesterol (HDL-C), LDL cholesterol (LDL-C) and triglyceride (TG)), glucose, insulin and glycated hemoglobin (HbA1c) concentrations were measured at the *Centre Hospitalier Universitaire de Québec* (CHUQ)-Université Laval. Total-C, HDL-C, TG and glucose were measured by enzyme-based assays. LDL-C was calculated with the Friedewald formula [27], and insulin was assessed by chemiluminescence. HbA1c was measured by ion exchange high-performance liquid chromatography.

Participants underwent oral glucose tolerance tests (OGTT) at the beginning (week 0) and at the end (week 8) of the supplementation period. Values for the fasting blood samples were calculated as the mean of times − 15 and 0 min before participants were asked to drink a 75-g glucose solution. Blood was further drawn at 15, 30, 60, 90 and 120 min after glucose solution intake. The homeostatic model assessment of insulin resistance (HOMA-IR) was computed as fasting glucose and fasting insulin product divided by the normalizing factor 22.5 [28]. Matsuda index [29] was calculated as 10,000 divided by square root of (Fasting glucose × Fasting insulin) × (Mean glucose × Mean insulin), with mean glucose and insulin concentrations being calculated as the mean of fasting, 30-, 60-, 90- and 120-min samples. Units of measurement were converted in order to comply with the units used for these equations. Due to high hemolysis in some blood samples, Matsuda index has been calculated for only a limited number of subjects in each group.

### Statistical analysis (MetS phenotype parameters)

SAS version 9.4 (SAS Institute, Cary, NC) was used for statistical analyses, which were by originally assigned groups. Each variable was assessed for normality using skewness and kurtosis. Non-normally distributed variables that have been transformed prior statistical analyses are indicated in footnotes below each table. Statistics are presented as means with standard deviations (SD) or ranges. The MIXED procedure in SAS (with compound symmetry as covariance structure) was used to test for differences in dietary intakes and indices of cardiometabolic health including HOMA-IR and Matsuda index. Treatment, time and treatment × time interaction were considered as fixed effects with repeated measurements. Post hoc comparisons among groups were performed using least square means (LS-means) when a significant treatment × time interaction (*p* < 0.05) was observed. Analyses were performed with and without adjustments for covariates including age, sex and BMI and/or nutrient intakes, as per the footnote of each table. A per-protocol analysis was used. *P* values < 0.05 were considered significant.

## Transcriptomics

Blood cell gene expression profiles were examined to assess nutrition-related metabolic changes occurring in response to the BBP. Blood samples were collected in PAXgene blood RNA tubes (Qiagen, Valencia, CA, USA) at week 0 and week 8, and were stored at − 80 °C until the analyses. Total RNA was extracted, and samples were sent to the *McGill University and Génome Québec Innovation Centre* for sequencing. The quality of RNA samples was evaluated with the 2100 Bioanalyzer (Agilent, Santa Clara, CA, USA). RNA samples were converted to cDNA with the Illumina NEB stranded mRNA library preparation kit (Illumina, San Diego, CA, USA; rRNA-depleted stranded (HMR)) for sequence library preparation based on the manufacturer’s protocol. Final libraries were sequenced on an Illumina NovaSeq6000 S4 sequencer using paired-end, 100 bp reads. For bioinformatic analyses, raw reads were trimmed for length (*n* = 50), quality (phred33 score ≥ 30) and adaptor sequence using Trim Galore (v0.6.5), a wrapper tool around Cutadapt (v1.15) and FastQC (v0.11.9). Trimmed reads were pseudo-aligned to the GRCh38 human reference transcriptome using kallisto (v0.46.2), and transcript abundance was estimated with default parameters and 100 bootstraps, and reported in estimated counts [30]. Data normalization and automatic filtering of estimated counts, as well as differential expression analysis were performed with edgeR v3.28.1 [31]. Given the paired nature of our samples, differential transcript expression across the BBP group from week 0 to week 8 was determined using a generalization of a paired *t* test implemented in the quasi-likelihood framework of edgeR. Differentially expressed transcripts between post- vs. pre-intervention with BBP were considered at a false-discovery rate (FDR)-corrected *P* value < 0.05. The functional significance of genes showing at least a 25% difference (1.25-fold change) and an unadjusted *P* value < 0.05 between pre- and post-supplementation states was explored by pathway enrichment analysis using the clusterProfiler v3.16.0 R package [32]. ClusterProfiler implements statistical methods to analyze and visualize functional profiles of genes/gene clusters and produces adjusted *P* values using the Benjamini-Hochberg procedure (BH-p) for significantly enriched pathways. The following pathway databases were used for functional enrichment analysis: Gene Ontology Biological Processes (GO-BP), Reactome and the Kyoto Encyclopedia of Genes and Genomes (KEGG). The cnetplot function implemented in the clusterProfiler package was used to visualize significantly enriched pathways.

## Metabolomics

Plasma samples collected at weeks 0 and 8 were sent to the Analytical Facility for Bioactive Molecules at the Hospital for Sick Children in Toronto, Canada. The quantitative analysis of 630 metabolites from 26 biochemical classes was performed in paired blood samples from 24 participants before and after the BBP supplementation with the MxP® Quant 500 kit for targeted metabolic profiling (Biocrates Life Sciences AG, Innsbruck, Austria). The analysis of metabolites combined flow injection analysis (FIA) with liquid chromatography-based triple quadrupole mass spectrometry (LC-MS/MS) and was performed in an Agilent 1200 series HPLC chromatograph coupled to a SCIEX QTrap 5500 mass spectrometer.

### Statistical analysis (metabolomics)

Metabolite data from 24 participants (pre- versus post-intervention with BBP) were processed using the MetaboAnalystR package (v3.0) [33]. First, 113 metabolites with a constant or single value across samples were found and deleted. Non-informative signals were further filtered out based on the interquartile range estimate, and samples were normalized by quantile normalization. Metabolite data were log-transformed and scaled by Pareto scaling (mean-centered and divided by the square root of the standard deviation of each variable). After quantile normalization, one additional metabolite with a constant value was found and deleted. From the original 630 metabolites analyzed, a total of 386 were included in the final statistical analysis. Paired *t* tests were used to analyze within-subject changes in blood metabolite levels between pre- and post-supplementation states. A volcano plot associated to paired *t* tests was further used to visualize the most differential metabolite changes between pre- and post-supplementation. A *P* value < 0.05, along with a count of significant pairs higher than 50% showing at least 25% difference (1.25-fold change), were the criteria used to consider metabolite blood levels to significantly differ between pre- and post-supplementation.

### Dimensional reduction

Dimensional reduction was conducted using the partial least squares discriminant analysis (PLS-DA), a supervised algorithm able to reduce the number of metabolites in high-dimensional metabolomics data to produce robust and easy-to-interpret models. This method is able to differentiate the class membership through multivariate regression of a given set of metabolites. In the present study, we used a variation of PLS-DA, the multilevel PLS-DA (mPLS-DA), given its ability to exploit the paired structure of the multivariate data obtained before and after the BBP supplementation in the same group of participants [34, 35]. A sparse mPLS-DA (smPLS-DA) was used to identify the most important metabolites that help discriminate matched study groups [36]. The smPLS-DA algorithm was implemented using the mixOmics R package (v6.12.1) [37]. The variable importance in projection (VIP) coefficients were computed as a weighted sum of squares of the smPLS-DA loadings to depict the relative importance of each metabolite in the classification model. Predictors with large VIP are the most relevant for discriminating class membership [37].

## Results

### Participants characteristics and adherence

**Fig. 2:**
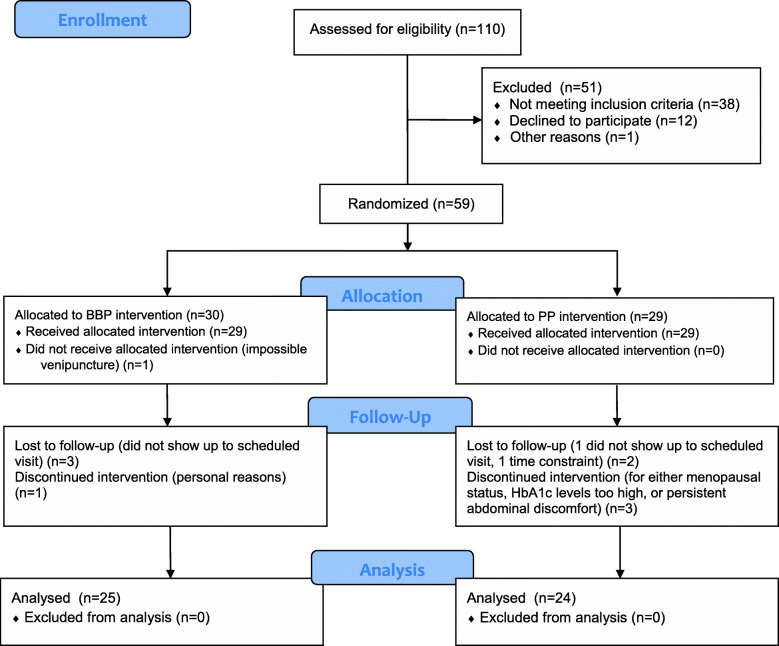
CONSORT 2010 Flow Diagram

Of the 110 individuals screened for eligibility, 59 participants were included in this randomized double-blind, placebo-controlled intervention trial, with analyses conducted on a final sample of 49 individuals who consumed the BBP (*n* = 25) or the placebo powder (*n* = 24) (Fig. 2). Characteristics of participants at week 0 who completed the study protocol are presented in Table 1. Participants were adults between the ages of 22 and 53. Adherence to the study protocol was robust, with an overall powder intake compliance rate of 92.7 ± 7.5% and no significant differences between groups (BBP: 92.4 ± 7.7% and placebo: (92.9 ± 7.3%). Results from the side effects questionnaire indicated that participants experienced some minor side effects with both the BBP and placebo powder (Supplementary Figure 1), but these side effects were generally tolerable with only one participant dropping out of the study as a result of persistent abdominal discomfort.

**Table 1:** General baseline characteristics of participants

	All subjects (***n*** = 49)	BBP (***n*** = 25)	Placebo (***n*** = 24)
**Weight (kg)**	91.5 [62.5–130.4]	89.6 [62.5–126.5]	93.6 [71.6–130.4]
**Height (cm)**	171.0 [152.5–190.3]	170.8 [152.5–187.8]	171.3 [156.0–190.3]
**BMI (kg/m**^**2**^**)**	31.3 [23.4–47.1]	30.8 [23.4–47.1]	31.8 [25.7–39.7]
**Waist circ. (cm)**	104.1 [81.0–131.3]	101.7 [81.0–131.3]	106.6 [82.6–127.2]
**Hip circ. (cm)**	111.8 [92.2–141.8]	110.0 [92.2–141.8]	113.6 [99.8–132.9]
**SBP (mmHg)**	115 [94–134]	115[99–132]	114 [94–134]
**DBP (mmHg)**	72 [52–91]	72 [57–82]	72 [52–91]
**Total-C (mmol/L)**	4.40 [2.56–6.49]	4.55 [2.99–6.41]	4.24 [2.56–6.49]
**TG (mmol/L)**	1.43 [0.36–3.98]	1.63 [0.53–3.98]	1.22 [0.36–2.30]
**HDL-C (mmol/L)**	1.18 [0.74–2.07]	1.16 [0.74–1.99]	1.21 [0.85–2.07]
**LDL-C (mmol/L)**	2.56 [1.09–4.21]	2.64 [1.58–4.05]	2.48 [1.09–4.21]
**Total-C/HDL-C**	3.87 [1.97–6.97]	4.09 [2.42–6.97]	3.65 [1.97–6.73]
**Fasting glucose (mmol/L)**^a^	4.9 [4.0–6.4]	5.02 [4.1–5.7]	4.83 [4.0–6.4]
**Fasting insulin (pmol/L)**^b^	82 [31–184]	80.9 [31–184]	82.4 [43–169]
**HbA1c (%)**	0.051 [0.045–0.058]	0.051 [0.045–0.056]	0.051 [0.045–0.058]
**Age (years)**	36.0 [22–53]	35.2 [23–53]	36.7 [22–48]
**Sex [*n*** **(% female)]**	27 (55)	13 (52)	14 (58)
**Highest education level completed [*n*** **(%)]**			
**High school** **College** **University**	4 (8) 16 (33) 29 (59)	3 (12) 6 (24) 16 (64)	1 (4) 10 (42) 13 (54)
**Occupation [n (%)]**			
**Student (full-time)** **Student (part-time)**	10 (20) 5 (10)	7 (28) 4 (16)	3 (13) 1 (4)
**Annual household income $CDN**^c^ **[n (%)]**			
**0–39,999** **40,000–79,000** **80,000–99,000** **≥ 100,000**	10 (21) 15 (32) 9 (19) 13 (28)	7 (29) 8 (33) 4 (17) 5 (21)	3 (13) 7 (30) 5 (22) 8 (35)

Values from weight through to age are raw means [range]

*Abbreviations*: *BMI*, body mass index; *Waist circ*, waist circumference; *Hip circ*, hip circumference; *SBP*, systolic blood pressure; *DBP*, diastolic blood pressure; *Total-C*, total cholesterol; *TG*, triglycerides; *HDL-C*, HDL cholesterol; *LDL-C*, LDL cholesterol; *HbA1c*, glycated hemoglobin; *BBP*, blueberry powder

^a^*n* = 48 (24 for BBP and 24 for placebo)

^b^*n* = 39 (21 for BBP and 18 for placebo)

^c^*n* = 47 (24 for BBP and 23 for placebo)

### Dietary intake

As presented in Table 2, participants had dietary intakes of approximately 2000 kcal/day. There were no significant between-treatment differences. Time effects were observed for carbohydrates, total sugars and fibre. These results relate to the nutritional content of the BBP and placebo powder, which are comprised of similar quantities of carbohydrates and total sugar with a higher fibre content in the BBP compared to the placebo powder (Supplementary Table 1); thus, these results relate to the compliance to the intervention protocols. There was a significant treatment × time interaction for fibre whereby the BBP group had significantly greater changes (increases) in fibre intake compared with the placebo group from week 0 to weeks 4 and 8.

**Table 2:** Effects of treatment and/or time on nutritional intake

	Week 0		Week 4		Week 8		***P*** value^**1**^		
Variable	BBP	Placebo	BBP	Placebo	BBP	Placebo	Treatment	Time	Treatment × Time
**Calories (kcal)**	1979 ± 598	2080 ± 550	2152 ± 553	2165 ± 479	2204 ± 664	2203 ± 540	0.67	0.07	0.68
**Carbohydrates (g)**	226 ± 71	242 ± 63	261 ± 60	269 ± 57	269 ± 75	279 ± 65	0.42	**0.0001**	0.89
**Total sugars (g)**	94 ± 30	103 ± 39	116 ± 28	122 ± 38	122 ± 39	128 ± 42	0.41	**< 0.0001**	0.94
**Fibre (g)**	20 ± 9^a^	24 ± 10^a^	31 ± 6^b^	21 ± 7^b^	30 ± 6^b^	24 ± 9	0.08	**< 0.0001**	**< 0.0001**
**Total fat (g)**	85 ± 31	87 ± 30	88 ± 30	84 ± 27	87 ± 33	86 ± 28	0.99	0.83	0.71
**Proteins (g)**	84 ± 25	93 ± 27	85 ± 26	92 ± 24	90 ± 33	89 ± 27	0.37	0.83	0.12
**Alcohol (g)**	3 ± 3	2 ± 2	3 ± 2	2 ± 2	3 ± 3	2 ± 2	0.15	0.65	0.35
**Vitamin C (mg)**	96 ± 38	118 ± 60	96 ± 25	98 ± 34	95 ± 28	112 ± 49	0.36	0.62	0.23

*Abbreviations*: *BBP* blueberry powder

Values are mean ± SD. ^1^ Unadjusted *P* value. Analyses were conducted using the MIXED procedure with SAS version 9.4. Vitamin C was log_10_ transformed. Significant *P* values are in bold. LSMEANS pairwise comparisons were performed when the main interaction factor effect was considered statistically significant (*P* < 0.05). Results that do not share the same letter (a, b) are significantly different (*P* < 0.05) from each other. BBP: *n* = 24 (week 0) and 23 (4 and 8 weeks); placebo: *n* = 22 (week 0) and 23 (4 and 8 weeks)

### MetS Phenotypes

Cardiometabolic risk factors at different time points during the intervention are presented in Table 3. A treatment effect was observed only for plasma TG levels. This difference was no longer significant after adjustments for age, sex, BMI and baseline values (data not shown). These results were also no longer significant after adjusting for calories, fibre or total sugar intake, individually, therefore suggesting that the addition of calories, fibre or total sugar from the interventions contributed to the results (data not shown). Treatment × time interactions were all non-significant.

**Table 3:** Indices of cardiometabolic health over time by intervention group

	Week 0		Week 4		Week 8		***P*** values		
	BBP *n* = 25	Placebo *n* = 24	BBP *n* = 25	Placebo *n* = 24	BBP *n* = 25	Placebo *n* = 24	Treatment	Time	Treatment × Time
**Weight (kg)**	89.6 ± 17.7	93.6 ± 15.3	90.3 ± 18.1	93.8 ± 15.4	90.1 ± 18.1	93.8 ± 15.4	0.44	**0.007**	0.39
**BMI (kg/m**^**2**^**)**	30.8 ± 6.2	31.8 ± 3.8	31.0 ± 6.3	31.9 ± 3.9	30.9 ± 6.3	31.9 ± 3.8	0.36	**0.02**	0.47
**Waist circ (cm)**	101.7 ± 13.3	106.6 ± 10.4	102.0 ± 14.5	106.5 ± 10.9	101.5 ± 14.4	107.0 ± 11.1	0.17	0.95	0.26
**SBP (mmHg)**	115 ± 9	114 ± 10	115 ± 8	113 ± 13	114 ± 8	114 ± 13	0.74	0.87	0.69
**DBP (mmHg)**	72 ± 7	72 ± 10	73 ± 7	70 ± 9	73 ± 7	73 ± 9	0.59	0.55	0.33
**Total-C (mmol/L)**	4.55 ± 1.03	4.24 ± 0.88	4.65 ± 1.02	4.39 ± 0.92	4.57 ± 0.97	4.32 ± 0.82	0.30	0.22	0.91
**TG (mmol/L)**	1.63 ± 0.96	1.22 ± 0.41	1.85 ± 0.96	1.30 ± 0.65	1.74 ± 0.77	1.35 ± 0.42	**0.02**	0.10	0.45
**HDL-C (mmol/L)**	1.16 ± 0.29	1.21 ± 0.28	1.16 ± 0.32	1.27 ± 0.30	1.15 ± 0.33	1.20 ± 0.24	0.18	0.08	0.19
**LDL-C (mmol/L)**	2.64 ± 0.81	2.48 ± 0.80	2.63 ± 0.70	2.52 ± 0.81	2.63 ± 0.80	2.51 ± 0.69	0.54	0.93	0.89
**Fasting glucose (mmol/L)**^a^	5.02 ± 0.43	4.83 ± 0.43	5.16 ± 0.43	4.95 ± 0.50	5.03 ± 0.31	4.94 ± 0.48	0.13	**0.04**	0.40
**Fasting insulin (pmol/L)**^b^	80.9 ± 33.4	82.4 ± 36.5	87.5 ± 66.7	81.0 ± 44.6	96.7 ± 47.8	95.9 ± 44.5	0.73	**0.02**	0.92
**HbA1C (%)**^c^	0.051 ± 0.003	0.051 ± 0.003	0.052 ± 0.003	0.050 ± 0.002	0.052 ± 0.003	0.051 ± 0.003	0.17	0.14	0.67

Results are presented as raw means ± SD. P-values are unadjusted. Analyses were conducted using the MIXED procedure with SAS version 9.4. Significant *P* values are in bold. The following non-normally distributed variables were log_10_ transformed: BMI and fasting insulin. HDL-C was inverse transformed

*Abbreviations*: *BMI* body mass index, *Waist circ* waist circumference, *SBP* systolic blood pressure, *DBP* diastolic blood pressure, *Total-C* total cholesterol, *TG* triglycerides, *HDL-L* HDL-cholesterol, *LDL-C* LDL-cholesterol, *HbA1c* glycated hemoglobin, *BBP* blueberry powder

^a^BBP: *n*=24 (week 0), *n*=25 (week 4 and 8); Placebo: *n*=24 (week 0 and 4), *n*=23 (week 8)

^b^BBP: *n*=21 (week 0), *n*=24 (week 4), *n*=22 (week 8); Placebo *n*=18 (week 0), *n*=24 (week 4), *n*=20 (week 8)

^c^BBP: *n*=25 (week 0 and 8), *n*=24 (week 4); Placebo *n*=24 (week 0, 4 and 8)

Changes from week 0 to week 8 in plasma concentrations of cardiometabolic risk factors were also investigated. As presented in Supplementary Table 2, changes were not significantly different between groups either in the unadjusted or the age, sex and BMI-adjusted models.

Data from the OGTT-derived markers of insulin resistance are presented in Table 4. For HOMA-IR, a threshold value of 2.5 is used to define individuals with IR [38]. A time effect was observed for HOMA-IR, with the mean being lower than the defined threshold value for BBP and placebo at week 0 but increased above the threshold after 8 weeks in both groups. For Matsuda index, a value of less than 4.3 predicts IR [38]. Only the placebo group had a mean value below this threshold at 8 weeks. After adjusting for calories, fibre or total sugar intake, individually, the time effect remained significant for Matsuda index (*P* < 0.04), was no longer significant for HOMA-IR (*P* > 0.05). No treatment × time interactions were revealed. All results remained unchanged after adjustment for age, sex and BMI (data not shown).

**Table 4:** Effects of treatment and/or time on insulin resistance (HOMA-IR) and insulin sensitivity (Matsuda index)

	Week 0		Week 8		P value		
	BBP	Placebo	BBP	Placebo	Treatment	Time	Treatment × Time
**HOMA-IR**^**1**^	2.38 ± 1.19	2.25 ± 0.93	2.64 ± 1.44	2.56 ± 1.15	0.80	**0.02**	0.53
**Matsuda index**^**2**^	4.86 ± 3.17	5.13 ± 2.80	4.28 ± 2.21	3.91 ± 2.19	0.71	**0.006**	0.08

Values are mean ± SD. Analyses were conducted using the MIXED procedure with SAS version 9.4. HOMA-IR and Matsuda index were non-normally distributed and were therefore log_10_ transformed. Abbreviations: *BBP* blueberry powder. Significant *P* values (unadjusted) are in bold. 1. BBP: *n* = 24 (week 0), *n* = 23 (week 8); placebo: *n* = 23 (week 0), *n* = 24 (week 8). 2. BBP: *n* = 18 (week 0), *n* = 17 (week 8); placebo: *n* = 17 (weeks 0 and 8)

### Transcriptomics: effect of gene expression

**Fig. 3:**
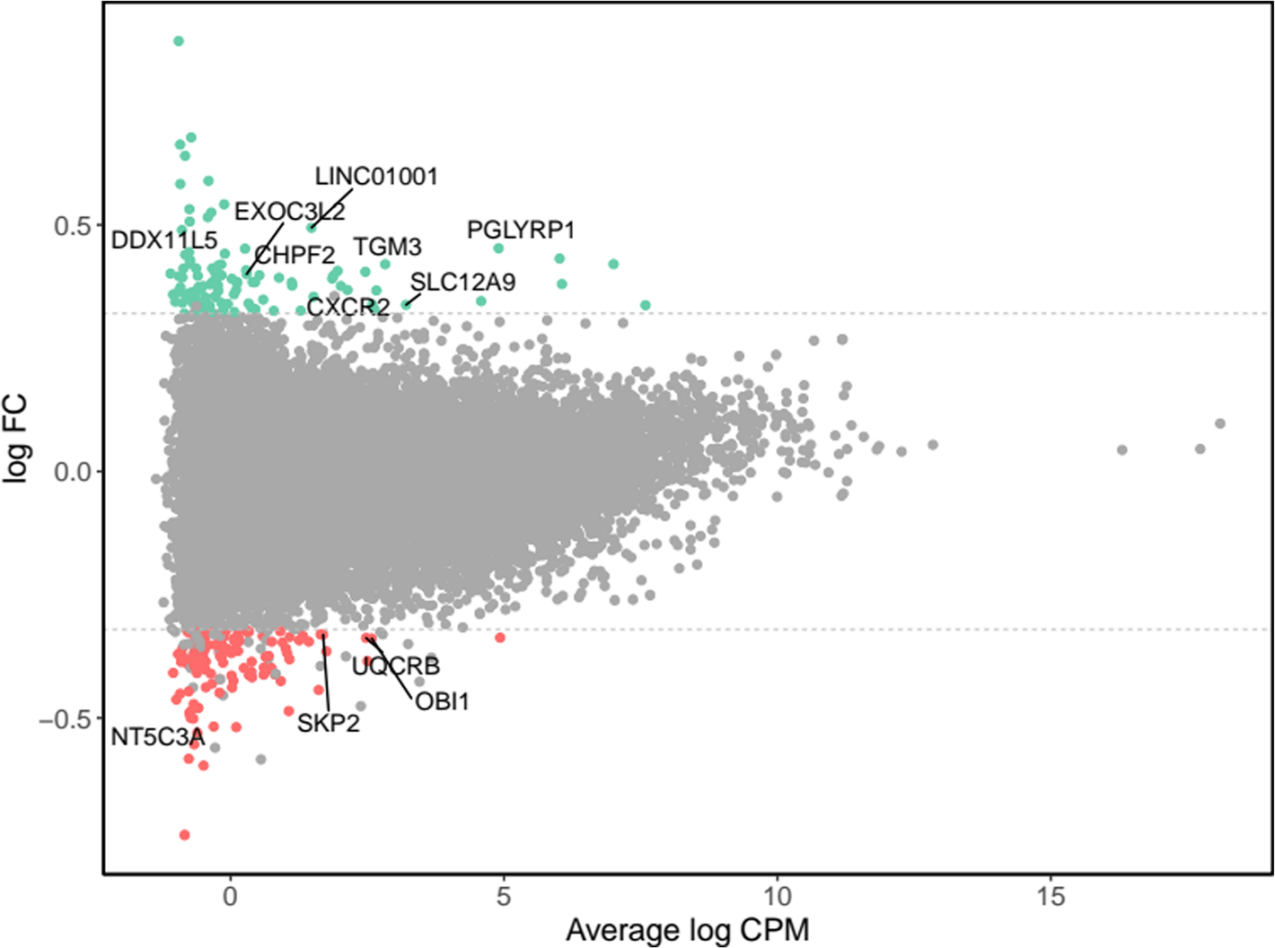
Global gene expression change between pre- and post-supplementation states in the blueberry group. MA plot shows the log2 average abundance of transcripts in counts per million mapped reads (log CPM) on the *x*-axis and the log2-fold change (log FC) on the *y*-axis. Non-significant genes are represented by grey dots. Over- and under-expressed genes (FC > 1.25) with unadjusted significant differences (paired *t* test *P* value < 0.05) are coloured in green and red, respectively. Significant differentially expressed genes from paired *t* tests (FDR-adjusted *P* value < 0.05) and showing at least a 1.25 FC are labelled with gene names. The dashed lines represent 1.25 FC

**Fig. 4:**
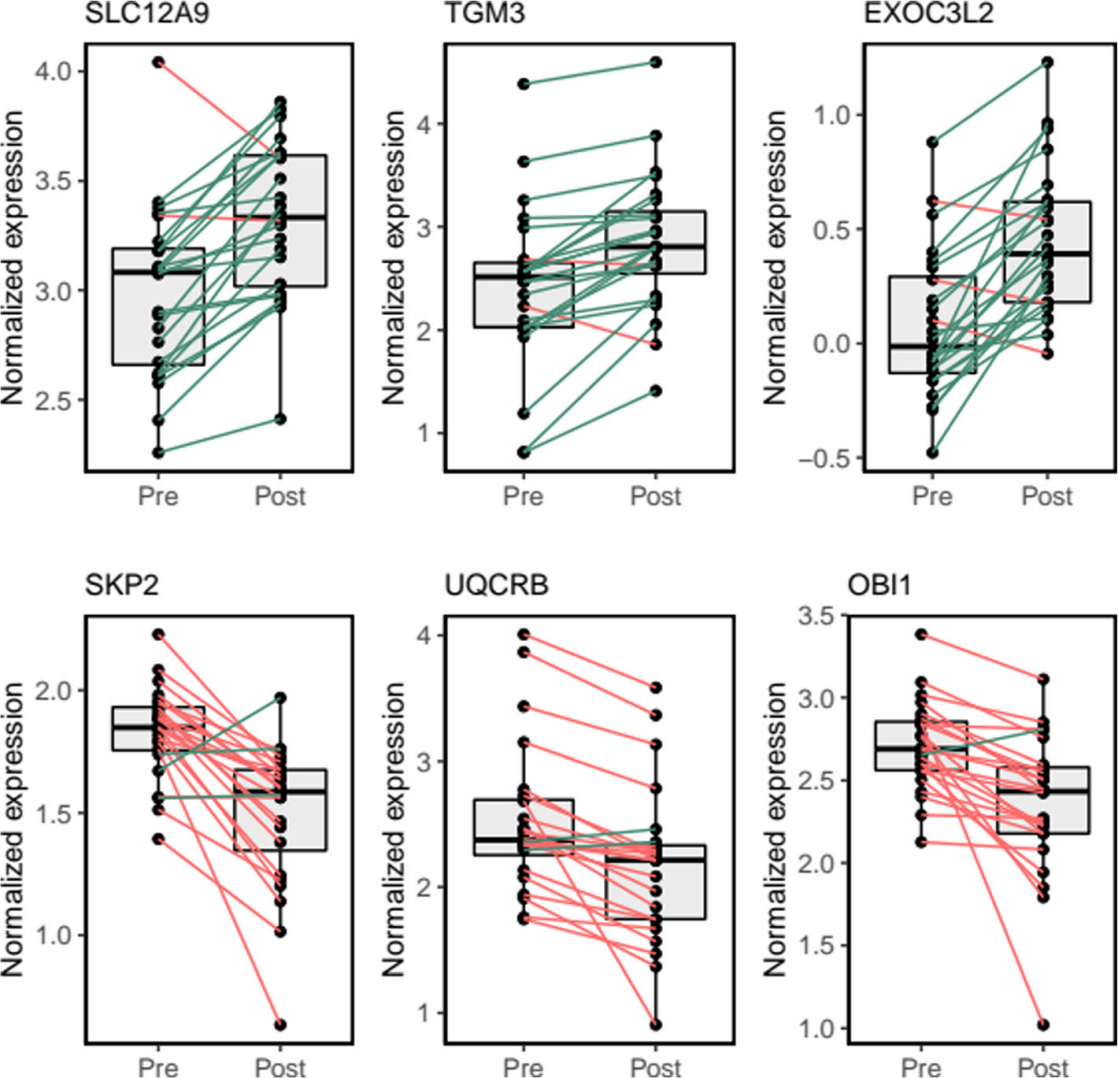
Top differentially expressed genes between pre- and post-supplementation states in the blueberry group. Box and whisker plots show median, first, and third quartiles, and maximum and minimum values for the 24 sample pairs before (Pre) and after (Post) the blueberry supplementation. The three transcripts which exhibited the most significant (FDR-adjusted *P* value < 0.05) over- and under-expression derived from paired *t* tests (Post vs Pre) are shown on the top and bottom rows, respectively. Green and red lines stand for increasing or decreasing gene expression levels between pre- and post-supplementation states within individual paired samples

**Fig. 5:**
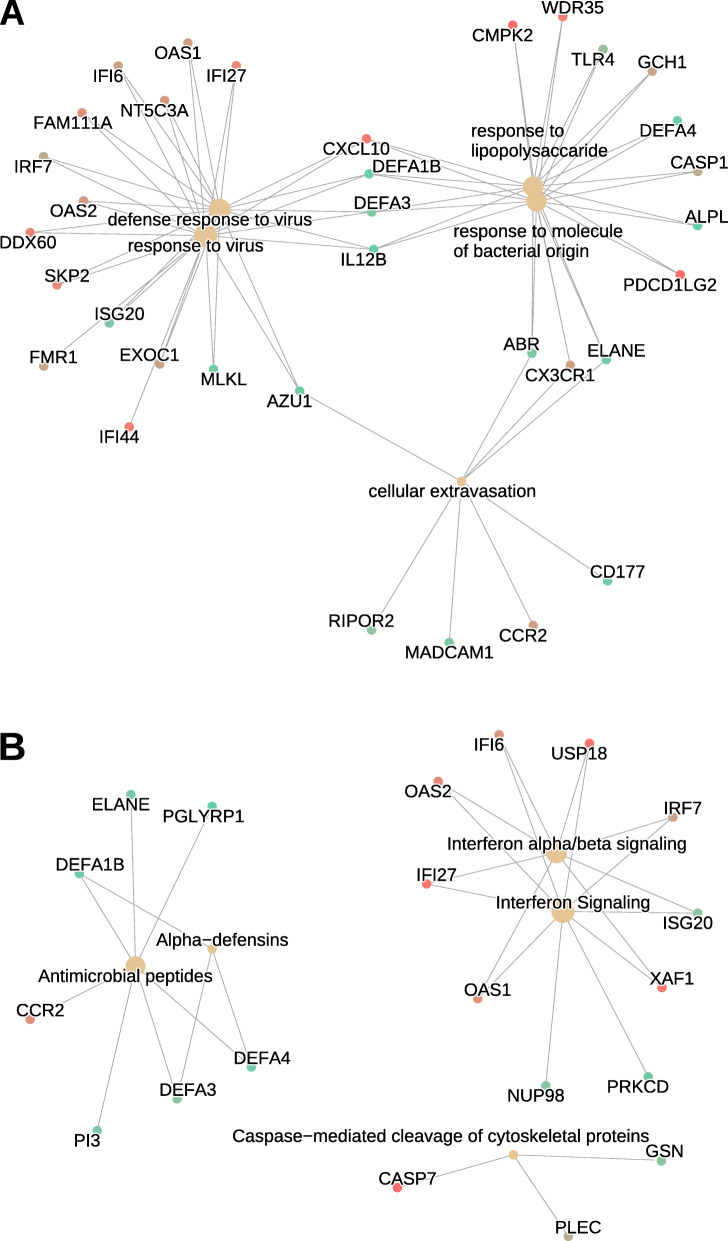
Network plots of enriched terms following the blueberry supplementation. The network plot depicts the linkages among differentially expressed gene clusters and functional enriched terms in the Gene Ontology Biological Processes (GO-BP) (**a**) and Reactome (**b**) pathway databases. The size of the grey dots is proportional to the number of genes in the enriched pathway (from 3 to 16) and the red-to-green color gradient of gene dots represents the direction of the gene expression fold change following the blueberry supplementation from down- to up-regulation, respectively

Results from the transcriptomics analyses are presented in Supplementary Table 3 and Figs. 3 and 4. The assessment of differential transcript expression revealed a total of 49 genes differentially expressed at FDR-adjusted *P* value < 0.05, which were differentially expressed from week 0 to week 8 in the BBP intervention group (Supplementary Table 3). Statistically significant fold changes in differentially expressed genes ranged from − 1.4 (*NT5C3A,* NM_001002010) to 1.4 (*DDX11L5,* NR_051986), with 12 genes showing at least a 1.25-fold change (Fig. 3). Individual changes in gene expression for the top differentially up- and downregulated genes are shown in Fig. 4. Pathway enrichment analysis revealed that the 259 most differentially expressed genes were clustered into immune-related pathways, as shown by the top-five significantly enriched GO-BP categories (Fig. 5a), including “defense response to virus” (*n* = 16 genes, BH-p = 3.0 × 10^-5^), “response to virus” (*n* = 18, BH-p = 3.7 × 10^-5^), “cellular extravasation” (*n* = 8, BH-p = 3.6 × 10^-4^), “response to lipopolysaccharide” (*n* = 15, BH-p = 3.2 × 10^-3^) and “response to molecule of bacterial origin” (*n* = 15, BH-p = 4.1 × 10^-3^). This is consistent with the five significantly enriched pathways found in the Reactome database (Fig. 5b), including “interferon alpha/beta signaling” (*n* = 8 genes, BH-p = 1.4 × 10^-3^), “interferon signaling” (*n* = 10, BH-p = 3.3 × 10^-2^), “antimicrobial peptides” (*n* = 7, BH-p = 3.3 × 10^-2^), “alpha defensins” (*n* = 3, BH-p = 3.3 × 10^-2^) and “caspase-mediated cleavage of cytoskeletal proteins” (*n* = 3, BH-p = 4.8 × 10^-2^). Finally, an immune-related KEGG pathway was also found to be significantly enriched, the “NOD-like receptor signaling pathway” (*n* = 10, BH-p = 9.8 × 10^-3^; network plot not shown).

### Metabolomics: effect of BBP on metabolites

**Fig. 6:**
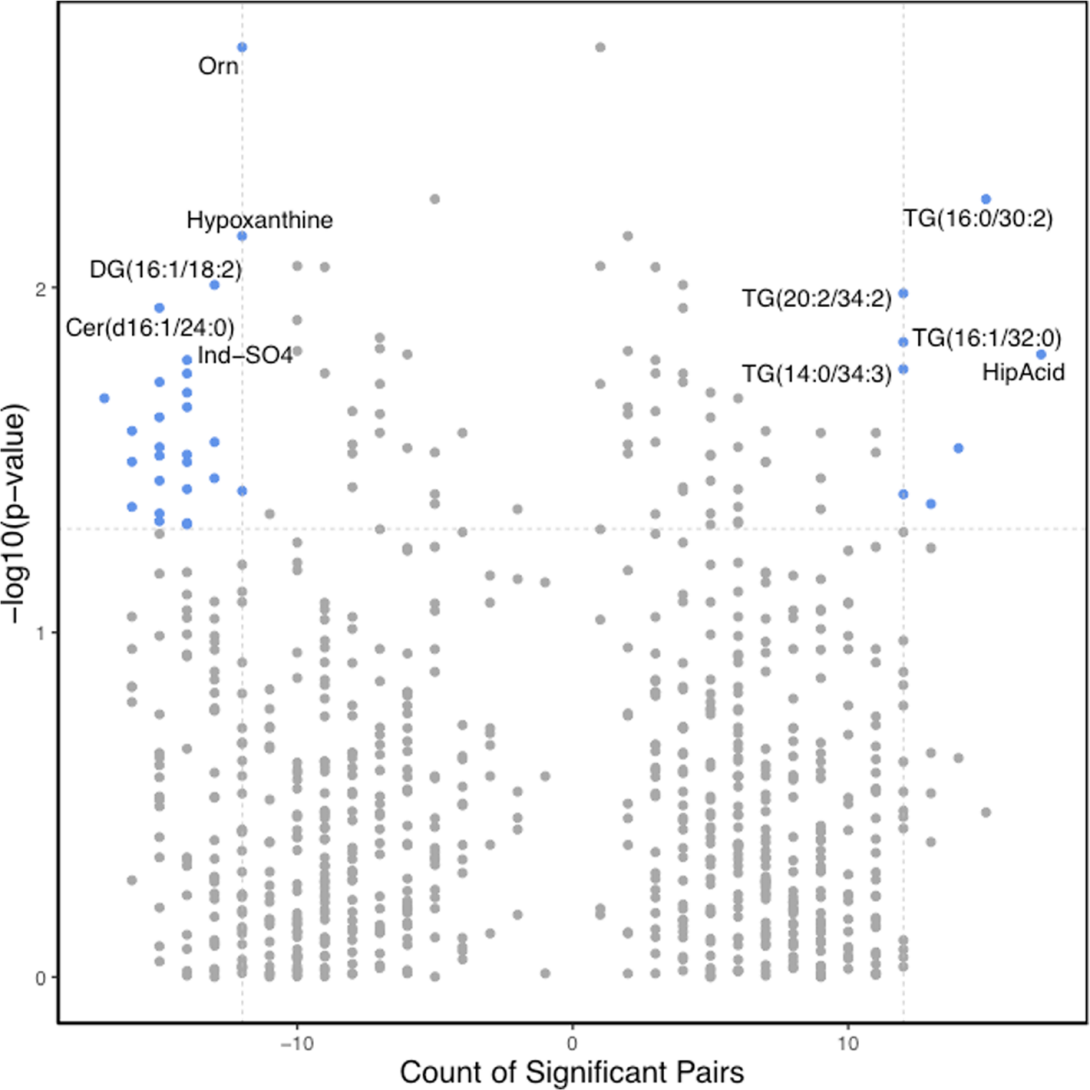
Impact of BBP supplementation on blood metabolite levels. Volcano plot of paired comparisons between metabolite blood levels in pre- and post-supplementation groups. On the *x*-axis, a count of significant sample pairs is shown. On the *y*-axis, the minus logarithm of paired *t* test *P* values is shown. Blood levels of a given metabolite were considered significantly different between pre- and post-supplementation states when the paired *t* test *P* value was < 0.05, the change in metabolite blood levels was higher than 25% (> 1.25-fold change), and the count of significant pairs was higher than the 50% of the total count of pairs. Each dot represents a metabolite. Metabolites showing statistically significant changes following the blueberry supplementation are depicted as blue dots on the right (increase) and left (decrease) top corners. Top-ten significantly different metabolites are labelled. *Orn* ornithine, *DG* diacylglycerol, *Cer* ceramide, *Ind-SO4* indoxyl sulfate, *TG* triglyceride, *HipAcid* hippuric acid

**Fig. 7:**
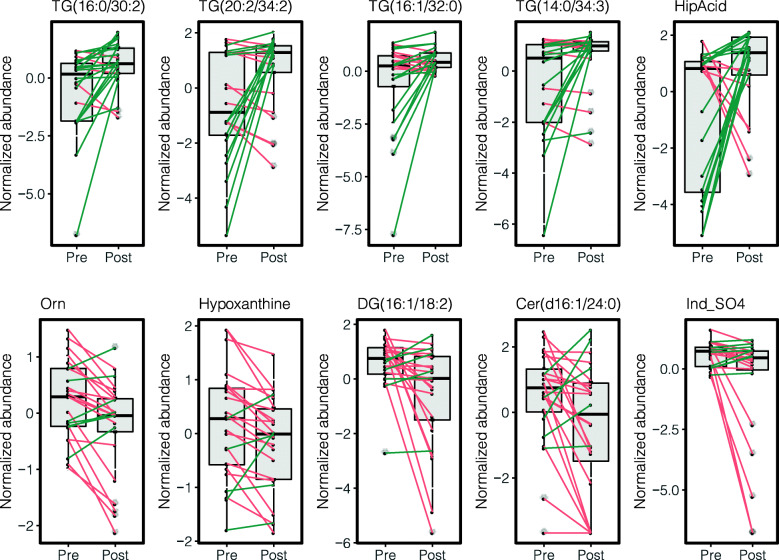
Top metabolites showing significant changes following BBP supplementation. Box and whisker plots show median, first, and third quartiles and maximum and minimum values for the 24 sample pairs before (Pre) and after (Post) the blueberry supplementation. The five metabolites which exhibited the most significant increases and decreases following the supplementation are shown on the top and bottom rows, respectively. Green and red lines stand for increasing or decreasing metabolite blood levels between pre- and post-supplementation states within individual paired samples. *Orn* ornithine, *DG* diacylglycerol, *Cer* ceramide, *Ind-SO4* indoxyl sulfate, *TG* triglyceride, *HipAcid* hippuric acid

The paired *t* test analysis demonstrated that following BBP supplementation, a total of 50 metabolites had significantly different blood levels (paired *t* test unadjusted *P* value < 0.05), as compared to pre-supplementation levels. This number was reduced to 35 metabolites after applying the statistical significance criteria (> 1.25-fold change and > 50% of significant metabolite counts) (Fig. 6 and Supplementary Table 4). None of the metabolites analyzed showed significant differences following multiple testing correction. Metabolites showing a significant reduction of their blood levels following the BBP supplementation are shown on the top-left corner of the volcano plot, and those showing a significant increase are shown on the top-right corner of the paired volcano plot (Fig. 6). The top-five under- and overabundant metabolites are shown in paired boxplots in Fig. 7. Among the under-abundant metabolites, those showing a more significant decrease are ornithine (Orn), hypoxanthine, diacylglycerol DAG(16:1/18:2), ceramide Cer(d16:1/24:0) and indoxyl sulfate (Ind-SO4). On the other hand, triglycerides TG(16:0/30:2), TG(20:2/34:2), TG(16:1/32:0), TG(14:0/34:3), TG(16:1/32:0) are among the metabolites that have undergone a significant increase following the BBP supplementation.

**Fig. 8:**
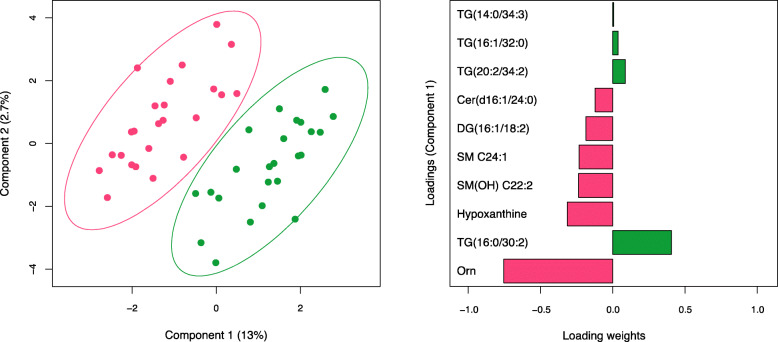
Identification of metabolites using Sparse multilevel partial least squares-discriminant analysis (smPLS-DA). A bi-dimensional score plot is shown on the left panel. The score plot reveals the distinct blood metabolomic profile between pre- (red dots) and post-supplementation (green dots) paired participants. The two principal components of the smPLS-DA model along with their corresponding variance in group discrimination are shown on *y*- and *x*-axes, respectively. The loading plot representing the top 10 metabolites selected on the first component of the smPLS-DA model is shown on the right. Horizontal bars represent the loading weights of each metabolite. Most important metabolites in group discrimination are ordered according to their loading weights, from bottom to top. Bar colour represents either an increase following supplementation (green bars) or decrease following supplementation (red bars). *TG* triglyceride, *Cer* ceramide, *DG* diacylglycerol, *SM* sphingolipid, *Orn* ornithine

Results from smPLS-DA strengthened findings from *t* test analysis, by identifying shared subsets of metabolites. The score plot derived from smPLS-DA shows the complete separation of pre- and post-supplementation groups, without overlap (Fig. 8). Component 1 was primarily responsible for group discrimination, accounting for a 13% of the variance, with component 2 accounting for 2.7%. The ten metabolites associated with the first component and underlying the discrimination between pre- and post-supplementation groups are shown in the loadings panel of the first component (Fig. 8). Most of these metabolites, whose relative contribution to group discrimination, or loading weight, is highlighted in Fig. 8, have been previously identified as differentially abundant between pre- and post-supplementation in the *t* test analysis (Fig. 7). The top five metabolites exhibiting significant reductions include ornithine, hypoxanthine, DAG(16:1/18:2), Cer(d16:1/24:0) and Ind-SO4. The top five metabolites exhibiting significant increases in response to BBP supplementation include TG(16:0/30:2), TG(20:2/34:2), TG(16:1/32:0), TG(14:0/34:3) and HipAcid.

## Discussion

This randomized placebo-controlled clinical trial investigated the effects of an 8-week BBP intervention on metabolic markers of cardiometabolic health, transcriptomics and metabolomics in adults at risk of developing MetS. Compared to placebo, BBP intake had no major, significant effect on cardiometabolic health or glycemic response after 8 weeks. However, transcriptomics and metabolomics data indicate significant changes occurring in response to BBP, thus demonstrating that BBP may have potential health-related effects after an intervention longer than 8 weeks and/or with whole blueberry consumption and/or a higher dosage.

Results from studies assessing the impact of highbush blueberries on health have been variable. With a 28-day supplementation of 11 g BBP daily, flow-mediated dilation improved and SBP was significantly reduced in men [39]. Similarly, a reduction in SBP and DBP was observed in a study of comparable size and identical duration and dose as in the present study, but composed mainly of women with the MetS [11]. However, with half of the BBP dose used by Basu et al. [11] and in the present study, no improvement in SBP or DBP following a 6-week BBP supplementation were observed in a sample of men [19]. Through a meta-analysis of studies with similar designs to the present RCT [40], the overall body of evidence suggests that highbush BBP does not have a significant effect on SBP (− 0.28 mmHg [95% CI: − 1.11, 0.56]) and DBP (− 0.50 mmHg [95% CI: − 1.24, 0.24]) [40]. It is however possible that whole blueberries have a significant effect on BP, as a meta-analysis of RCTs reported a favourable effect of 2- to 24-week duration of supplementation with whole berries (including studies on cranberries, bilberries, blueberries, whortleberries, elderberries or raspberries), on SBP (− 2.72 mmHg [95% CI: − 5.32, − 0.12]) [41].

In contrast to findings made in human interventions, numerous animal studies reported benefits of blueberries alone or mixed with other berries on weight management [42–44], markers of glucose/insulin resistance or sensitivity [10, 42–46], lipid profile [42, 47, 48], vascular health [49, 50], kidney function [51] or inflammatory markers [10, 42–44, 50–53]. Results from studies assessing other MetS phenotype parameters have been consistent with our findings overall. Using a similar design and intervention protocol, Basu and colleagues reported no difference between the placebo and the blueberry group for serum glucose concentration, lipid profile, HbA1c and HOMA-IR [11]. Similarly, in a study providing blueberry smoothies for a 6-week period, no impact on lipid, fasting glucose and insulin levels or insulin sensitivity were observed [18]. In these two clinical trials, obese participants with MetS were recruited and similar dose of BBP were given as in the present study. Moreover, a meta-analysis reported no effect of berry supplementation on total-C, HDL-C and TG levels, but the berry supplementation did lead to a significant reduction in LDL-C (− 0.21 mmol/L [95% CI: − 0.34, − 0.07]), fasting glucose (− 0.10 mmol/L [95% CI: − 0.17, − 0.03]), HbA1c [− 0.20% [95% CI: − 0.39, − 0.01]) and BMI (− 0.36 kg/m^2^ [95% CI: − 0.54, − 0.18]) [41]. Results reported herein are comparable to those reported in previous studies and in a meta-analysis including healthy and metabolically deteriorated individuals [40]. It is also important to keep in mind that although at least one cardiometabolic parameter was improved in most human intervention studies with BBP (e.g. blood pressure or inflammation biomarkers), the effect size remained quite small. Moreover, variability in baseline cardiometabolic parameters may contribute to differing results. For example, in the present study baseline BP was within the normal range overall and no significant BP changes were observed, but in a sample of participants with hypertension, BBP may be more likely to affect BP.

Additionally, fasting insulin and glucose levels are limited measures of insulin sensitivity. The gold standard for its assessment is the euglycemic clamp but because it is a strenuous procedure, 120- or 180-min OGTTs are more often used to get information on body glucose disposal efficiency after a test meal or a glucose load [54] from which various indices have been derived to get a better idea of glucose and insulin dynamics [38, 54]. One of them, HOMA-IR, is very common and the simplest to compute [28]. Another, the Matsuda index, is a good approximation of whole-body insulin sensitivity [29, 54]. Many studies have reported on these outcomes in response to blueberry interventions. Similar to the results presented herein, a 12-week tart cherry juice supplementation had no effect on HOMA-IR or insulin concentration [55]. Furthermore, a meta-analysis of pomegranate supplementation reported no change on mean HOMA-IR with a pooled estimated effect of − 0.04 [95% CI: − 0.53, 0.46] [56]. Moreover, a red wine polyphenol supplementation study for 8 weeks did not improve HOMA-IR, fasting glucose and insulin concentrations nor Matsuda index following a mixed-meal test in obese participants [57].

A possible explanation for the absence of effect of the BBP on cardiometabolic risk factors in the present study may be attributable to the extra carbohydrates and sugars provided by the powders, being added to the usual diet of study participants. While the analysis of energy intake did not reveal significant changes between baseline and treatment weeks, participants of both groups consumed significantly more carbohydrates at weeks 4 and 8 in comparison to week 0, of which about two third were sugars. Higher carbohydrate intake has been associated with lower HDL-C and higher TG concentrations [58]. The net increase in the intake of carbohydrates from the powders might have masked the potential lowering effects of BBP antioxidants on cardiometabolic parameters if the participants did not compensate by reducing their consumption of carbohydrates from other foods and beverages. In addition, the heterogeneity of participants whereby some had elevated TG, some had elevated fasting insulin and others had both high TG and high insulin levels may have obscured the results and reduced the probability of detecting significant associations. We cannot rule out the possibility that cardiometabolic parameters are differentially affected depending on an individual’s profile at the entry in the study; this should be further investigated.

The transcriptomics and metabolomics analyses allowed for the identification and understanding of activated metabolic pathways following the BBP dietary intervention. BBP had a significant impact on 35 individual metabolites and the expression of 49 genes, thus providing other important insights for future research exploring health-related outcomes resulting from BBP supplementation. It is possible that BBP had an effect on markers of inflammation, immunity and oxidative stress, but these outcomes were not investigated in the present study. Further exploration of the roles of each of these metabolites and genes can help guide future research endeavours. As a targeted metabolomics profiling, we acknowledge that this study is restricted to a limited number of metabolites and biochemical classes; although the targeted profile was comprehensive, exploring microbiota-derived metabolites other than secondary bile acids, indoles or branched-chain amino acids would have been of interest to the present study. Nevertheless, some interesting findings are worth highlighting. For example, ornithine, produced via the urea cycle, is a non-essential amino acid [59]. The substrate action of ornithine can lead to excessive polyamine synthesis which plays a role in modulating the development of certain types of cancer [59, 60]. This protective role has been supported by previous, *in vitro,* preclinical and clinical research on the effects of blueberries and other berries [61]. Indeed, future research is needed to explore this further and investigate the metabolic relevance of a reduction in plasma ornithine concentrations following blueberry supplementation. There was also a significant reduction in hypoxanthine following BBP supplementation. Circulating concentrations of hypoxanthine, a purine molecule which is a by-product of adenosine triphosphate catabolism, are elevated following an ischemic event [62]. On the other hand, the increase in plasma hippuric acid, we observed has been previously reported following anthocyanin supplementation [20, 63–65]. The significant increases in several TG metabolites following BBP supplementation was, to our knowledge, a novel finding. However, this finding was perhaps not surprising given the well-established relationship between carbohydrate intake and plasma TG [66]. Therefore, this finding of increased TG metabolites further relates to our dietary results demonstrating a significant increase in carbohydrates and total sugars throughout the intervention (due to the nutritional breakdown of the intervention products: Supplementary Table 1). While previous research has demonstrated that blueberries play a role in combatting oxidative stress [11], we did not observe significant changes in gene expression or metabolites related to oxidative stress pathways or 8-iso-PGF2α plasma levels [67].

Pathway analyses can determine if differentially expressed genes are part of predefined physiological networks more than what would be expected by chance alone. This allows for the generation of mechanistic hypotheses and identification of putative mechanisms [68]. It was interesting to find that differentially regulated genes were clustered into immune-related pathways, thus suggesting that the BBP supplementation could have anti-inflammatory effects. This finding is consistent with a formerly conducted transcriptomic analysis following a 4-week, 1L/day blueberry-apple juice dietary intervention, whereby the researchers also found gene expression changes in immune-response pathways alongside signalling pathways for apoptosis, cell adhesion and lipid metabolism [69]. This further relates to previous research indicating that blueberries can have immunomodulatory effects and reduce oxidative stress in adults with MetS [70], and that a blueberry green tea polyphenol soy complex could have a potential protective role against viral infections in athletes [71]. Moreover, a randomized controlled trial of 38 g/day BBP for 6 weeks resulted in a significant increase in natural killer cells, a type of peripheral lymphocyte playing a key role in the immune response [72]. It is important to note that our analysis was performed in whole blood samples, with gene expression analysis being consequently carried out on circulating immune cells, which are the most relevant cells to the immune system [73] and thus differential gene expression related to immunity may be expected. However, previous research has demonstrated that white blood cells, mainly peripheral blood mononuclear cells, adequately reflect the expression of the majority of genes in a metabolically relevant tissue such as the skeletal muscle tissues [74]. The identified changes in key molecular signalling pathways and metabolic regulatory networks can ultimately be used to identify relevant biological and metabolic pathways that can become targets for therapy and may assist researchers in developing new hypotheses related to the health benefits of blueberries. However, although it is also possible that a nutritional intervention may alter the composition of different cell types in blood, which then causes the changes in gene expression, the BBP intervention did not seem to induce any significant change in immune cell count (data not shown).

Taken together, while BBP did not appear to impact specific cardiometabolic risk factors related to MetS within the given time frame of the study, the significant changes in gene expression and metabolites indicate the potential for BBP to impact various health outcomes, particularly those related to immunity. MetS-related health outcomes may occur beyond an 8-week intake period or from an intervention with whole blueberries rather than BBP or a higher dosage. For example, a long-term BBP supplementation study in individuals with MetS investigated the benefits of taking 13 g or 22 g BBP on a daily basis (representing respectively a quarter and a half of the dose given in the present study), for a period of 6 months found significant improvements of endothelial function and arterial stiffness in subjects consuming the highest dose of BBP [20].

The present study had some limitations. First, it included premenopausal women, but menstrual cycle hormonal fluctuation can influence cardiometabolic biomarkers especially those associated to the lipid profile [75] and endothelial function [76]. Since the only constraint regarding powder intake was not to heat it, it is possible that consuming it with food such as milk could have had an effect on its antioxidant properties due to the matrix effect [77]. Furthermore, milled freeze-dried highbush BBP was used as a surrogate of whole fresh blueberries for practical reasons. Although this method has been reported to preserve blueberry antioxidants [78, 79], we cannot discard the possibility that fresh blueberries may exert more potent cardiometabolic benefits than those freeze-dried and milled into powder. Nonetheless, the strengths of the present study include its randomized, placebo-controlled, double-blind design that limits the influence of confounding factors, which may bias estimates of treatment effects. Compliance to treatment was high and study directives were well followed by participants. However, given the overall significant increase in carbohydrates and total sugars over time, it is not possible to differentiate between an independent BBP effect or an overall macronutrient effect on metabolic parameters within the present study. Powders were of similar aspect and taste and, unless comparing BBP and placebo powder side to side, participants were probably unable to discover to which treatment they were randomized. Also, it is important to highlight that blueberry supplementation studies in healthy but overweight individuals at risk of developing MetS are less frequent thus highlighting the novelty of this work. Most studies have been conducted on subjects older than those in the present study and/or having a MetS diagnosis. Moreover, in addition to reporting changes for traditional biomarkers of cardiometabolic health, this trial also includes analyses on glucose and insulin resistance/sensitivity indices, which are better indicators than fasting values alone. The literature was especially lacking human interventions reporting these measurements. Lastly, to our knowledge, this was the first study to assess changes in gene expression resulting from BBP supplementation. Finally, this study is primarily generalizable to men and women at risk of MetS.

## Conclusion

In conclusion, no significant effects on plasma cardiometabolic risk factors were observed herein following an 8-week intervention with BBP. However, given the significant changes in gene expression and several metabolites following BBP supplementation, it is plausible that a longer follow-up and/or higher dosage could have resulted in significant improvements in plasma MetS parameters. Future BBP interventional clinical trials should seek to explore health outcomes related to the changes in gene expression and metabolites reported in the current trial, while providing an intervention > 8 weeks, intervening with whole blueberries, and/or providing a higher dosage of BBP.

## Supplementary Information

**Additional file 1: Supplementary Table 1**. Nutrition Facts: Blueberry Powder and Placebo Powder

**Additional file 2: Supplementary Figure 1**. Proportion of participants who experienced side effects following powder consumption

**Additional file 3: Supplementary Table 2**. Effect of treatment on changes in cardiometabolic variables of interest

**Additional file 4: Supplementary Table 3**. Transcriptomics results for significant (*p*<0.05) changes in gene expression from week 0 to week 8 in BBP group

**Additional file 5: Supplementary Table 4**. Metabolomics results for significant (*p*<0.05) changes in metabolites from week 0 to week 8 in BBP group

## Data Availability

Please contact authors for data requests.

## Abbreviations

BBP: Blueberry powder
BMI: Body mass index
DBP: Diastolic blood pressure
FFQ: Food frequency questionnaire
IR: Insulin resistance
MetS: Metabolic syndrome
ROS: Reactive oxygen species
SBP: Systolic blood pressure
TG: Triglycerides
