# Supplementary material for: An 8-week freeze-dried blueberry supplement impacts immune-related pathways: a randomized, double-blind placebo-controlled trial
Source: Genes Nutr. 2021 May 17;16:7. doi: 10.1186/s12263-021-00688-2 (PMC8130140; doi:10.1186/s12263-021-00688-2)
Supplement: Supplementary file 1 — Additional file 1: Supplementary Table 1. Nutrition Facts: Blueberry Powder and Placebo Powder [file 12263_2021_688_MOESM1_ESM.docx]

**SUPPLEMENTARY TABLE 1**

**Nutrition Facts: Blueberry Powder**

| **Per 50g**  **Nutrients** | **Amount** |
| --- | --- |
|  |  |
| Calories | 197 kcal |
| Fat | 0 g |
| Sodium | 2 mg |
| Carbohydrates | 48 g |
| Fibre | 13 g |
| Sugars | 31 g |
| Protein | 1 g |
| Vitamin C | 11 mg |
| Potassium | 215 mg |

**Nutrition Facts: Placebo Powder**

| **Per 50g**  **Nutrients** | **Amount** |
| --- | --- |
|  |  |
| Calories | 195 kcal |
| Fat | 0 g |
| Sodium | 10 mg |
| Carbohydrates | 49 g |
| Fibre | 1 g |
| Sugars | 30 g |
| Protein | 0 g |
| Vitamin C | 2 mg |
| Potassium | 10 mg |
