# Supplementary material for: An 8-week freeze-dried blueberry supplement impacts immune-related pathways: a randomized, double-blind placebo-controlled trial
Source: Genes Nutr. 2021 May 17;16:7. doi: 10.1186/s12263-021-00688-2 (PMC8130140; doi:10.1186/s12263-021-00688-2)
Supplement: Supplementary file 2 — Additional file 2: Supplementary Figure 1. Proportion of participants who experienced side effects following powder consumption [file 12263_2021_688_MOESM2_ESM.docx]

## SUPPLEMENTARY FIGURE 1

**Proportion of participants who experienced side effects following powder consumption**
