# Supplementary material for: An 8-week freeze-dried blueberry supplement impacts immune-related pathways: a randomized, double-blind placebo-controlled trial
Source: Genes Nutr. 2021 May 17;16:7. doi: 10.1186/s12263-021-00688-2 (PMC8130140; doi:10.1186/s12263-021-00688-2)
Supplement: Supplementary file 3 — Additional file 3: Supplementary Table 2. Effect of treatment on changes in cardiometabolic variables of interest [file 12263_2021_688_MOESM3_ESM.docx]

**SUPPLEMENTARY TABLE 2: Effect of treatment on changes in cardiometabolic variables of interest**

|  | **Delta change** | | ***p*-value** | |
| --- | --- | --- | --- | --- |
|  | **BBP**  (n=25) | **Placebo**  (n=24) | **Unadjusted** | **Adjusted*** |
| **Weight** | 0.50 ± 1.17 | 0.28 ± 1.43 | 0.54 | 0.57 |
| **BMI** | 0.17 ± 0.41 | 0.09 ± 0.49 | 0.51 | 0.53 |
| **Waist circ** | -0.18 ± 1.76 | 0.36 ± 2.90 | 0.44 | 0.46 |
| **SBP** | -0.51 ± 6.79 | -0.24 ± 6.36 | 0.89 | 0.91 |
| **DBP** | 0.55 ± 5.21 | 0.35 ± 4.97 | 0.89 | 0.83 |
| **Total-C** | 0.02 ± 0.53 | 0.08 ± 0.48 | 0.69 | 0.55 |
| **TG** | 0.11 ± 0.63 | 0.13 ± 0.33 | 0.85 | 0.74 |
| **HDL-C** | -0.02 ± 0.11 | -0.01 ± 0.16 | 0.85 | 0.91 |
| **LDL-C** | -0.01 ± 0.47 | 0.03 ± 0.41 | 0.75 | 0.65 |
| **Fasting glucose^1^** | 0.00 ± 0.32 | 0.12 ± 0.33 | 0.22 | 0.16 |
| **Fasting insulin^2^** | 8.2 ± 21.7 | 16.8 ± 32.8 | 0.45 | 0.46 |
| **HbA1c** | 0.0005 ± 0.0014 | 0.0002 ± 0.0016 | 0.54 | 0.70 |
| Results are presented as raw delta change means ± SD. Analyses were made using the MIXED procedure with SAS studio v.3.8. **p*-value adjusted for age and sex for weight, BMI and waist circ or age, sex and BMI for other variables.   1. BBP: n=24; Placebo: n=23 2. BBP: n=19; Placebo: n=17   Abbreviations: BMI: body mass index; waist circ: waist circumference; SBP: systolic blood pressure; DBP: diastolic blood pressure; Total-C: total-cholesterol; TG: triglycerides; HDL-C: HDL-cholesterol; LDL-C: LDL-cholesterol; HbA1c: Glycated hemoglobin; BBP: blueberry powder.  The MIXED procedure was performed with delta changes calculated with log_10_ transformed values for fasting insulin at week 0 and 8. | | | | |
