# Supplementary material for: An 8-week freeze-dried blueberry supplement impacts immune-related pathways: a randomized, double-blind placebo-controlled trial
Source: Genes Nutr. 2021 May 17;16:7. doi: 10.1186/s12263-021-00688-2 (PMC8130140; doi:10.1186/s12263-021-00688-2)
Supplement: Supplementary file 4 — Additional file 4: Supplementary Table 3. Transcriptomics results for significant (p<0.05) changes in gene expression from week 0 to week 8 in BBP group [file 12263_2021_688_MOESM4_ESM.docx]

**Supplementary Table 3: Transcriptomics results for significant (*p*<0.05) changes in gene expression from week 0 to week 8 in BBP group**

| **Accession Number** | **FC** | **q-value** | **Gene Symbol** | **Gene Name** |
| --- | --- | --- | --- | --- |
| NR_051986 | 1.42 | 0.049 | *DDX11L5* | DEAD/H-box helicase 11 like 5 |
| NR_028326 | 1.40 | 0.030 | *LINC01001* | long intergenic non-protein coding RNA 1001 |
| NM_005091 | 1.37 | 0.025 | *PGLYRP1* | peptidoglycan recognition protein 1 |
| NM_003245 | 1.34 | 0.014 | *TGM3* | transglutaminase 3 |
| NM_138568 | 1.32 | 0.020 | *EXOC3L2* | exocyst complex component 3 like 2 |
| NM_001284295 | 1.31 | 0.026 | *CHPF2* | chondroitin polymerizing factor 2 |
| NM_001363494 | 1.27 | 0.014 | *SLC12A9* | solute carrier family 12 member 9 |
| NM_001168298 | 1.26 | 0.034 | *CXCR2* | C-X-C motif chemokine receptor 2 |
| NM_001319196 | 1.24 | 0.042 | *S100A8* | S100 calcium binding protein A8 |
| NM_000904 | 1.21 | 0.027 | *NQO2* | N-ribosyldihydronicotinamide:quinone reductase 2 |
| NM_000804 | 1.21 | 0.038 | *FOLR3* | folate receptor gamma |
| NR_033429 | 1.21 | 0.038 | *ABTB1* | ankyrin repeat and BTB domain containing 1 |
| NM_001329425 | 1.21 | 0.049 | *RGL4* | ral guanine nucleotide dissociation stimulator like 4 |
| NM_001320458 | 1.21 | 0.049 | *WNT9B* | Wnt family member 9B |
| NM_001320605 | 1.21 | 0.026 | *ICAM3* | intercellular adhesion molecule 3 |
| NM_001286447 | 1.21 | 0.039 | *RIPOR2* | RHO family interacting cell polarization regulator 2 |
| NM_001319201 | 1.21 | 0.049 | *S100A8* | S100 calcium binding protein A8 |
| NM_205837 | 1.20 | 0.020 | *LST1* | leukocyte specific transcript 1 |
| NM_148962 | 1.19 | 0.020 | *OXER1* | oxoeicosanoid receptor 1 |
| NM_005451 | 1.19 | 0.042 | *PDLIM7* | PDZ and LIM domain 7 |
| NM_013416 | 1.18 | 0.030 | *NCF4* | neutrophil cytosolic factor 4 |
| NM_001294333 | 1.17 | 0.020 | *ATP6V0B* | ATPase H+ transporting V0 subunit b |
| NM_031925 | 1.17 | 0.028 | *TMEM120A* | transmembrane protein 120A |
| NM_000578 | 1.17 | 0.034 | *SLC11A1* | solute carrier family 11 member 1 |
| NM_002756 | 1.17 | 0.049 | *MAP2K3* | mitogen-activated protein kinase kinase 3 |
| NM_001318325 | 1.16 | 0.047 | *NUP214* | nucleoporin 214 |
| NR_028325 | 1.16 | 0.049 | *LOC100132062* | uncharacterized LOC100132062 |
| NM_001142573 | 1.16 | 0.049 | *IMPDH1* | inosine monophosphate dehydrogenase 1 |
| NM_001785 | 1.15 | 0.030 | *CDA* | cytidine deaminase |
| NM_031968 | 1.15 | 0.038 | *NARF* | nuclear prelamin A recognition factor |
| NM_002455 | 1.14 | 0.030 | *MTX1* | metaxin 1 |
| NM_006755 | 1.14 | 0.049 | *TALDO1* | transaldolase 1 |
| NM_004037 | 1.13 | 0.049 | *AMPD2* | adenosine monophosphate deaminase 2 |
| NM_002746 | 1.12 | 0.049 | *MAPK3* | mitogen-activated protein kinase 3 |
| NM_207354 | 1.11 | 0.034 | *ANKRD13D* | ankyrin repeat domain 13D |
| NR_135579 | 1.11 | 0.049 | *ZNF652* | zinc finger protein 652 |
| NM_014597 | -1.10 | 0.041 | *DNTTIP2* | deoxynucleotidyltransferase terminal interacting protein 2 |
| NM_004094 | -1.13 | 0.020 | *EIF2S1* | eukaryotic translation initiation factor 2 subunit alpha |
| NM_018471 | -1.13 | 0.030 | *ZC3H15* | zinc finger CCCH-type containing 15 |
| NM_001079864 | -1.13 | 0.049 | *TAX1BP1* | Tax1 binding protein 1 |
| NM_018164 | -1.15 | 0.030 | *INTS13* | integrator complex subunit 13 |
| NM_018229 | -1.15 | 0.041 | *AP5M1* | adaptor related protein complex 5 subunit mu 1 |
| NM_003142 | -1.16 | 0.030 | *SSB* | small RNA binding exonuclease protection factor La |
| NM_032383 | -1.16 | 0.049 | *HPS3* | HPS3 biogenesis of lysosomal organelles complex 2 subunit 1 |
| NM_174916 | -1.21 | 0.049 | *UBR1* | ubiquitin protein ligase E3 component n-recognin 1 |
| NM_005983 | -1.26 | 0.020 | *SKP2* | S-phase kinase associated protein 2 |
| NM_006294 | -1.27 | 0.020 | *UQCRB* | ubiquinol-cytochrome c reductase binding protein |
| NM_024546 | -1.27 | 0.028 | *OBI1* | ORC ubiquitin ligase 1 |
| NM_001002010 | -1.44 | 0.041 | *NT5C3A* | 5'-nucleotidase, cytosolic IIIA |

FC indicates the gene expression fold change from week 0 to week 8. q-value stands for False Discovery Rate (FDR)-adjusted p-values from paired t-tests.
