# Supplementary material for: An 8-week freeze-dried blueberry supplement impacts immune-related pathways: a randomized, double-blind placebo-controlled trial
Source: Genes Nutr. 2021 May 17;16:7. doi: 10.1186/s12263-021-00688-2 (PMC8130140; doi:10.1186/s12263-021-00688-2)
Supplement: Supplementary file 5 — Additional file 5: Supplementary Table 4. Metabolomics results for significant (p<0.05) changes in metabolites from week 0 to week 8 in BBP group [file 12263_2021_688_MOESM5_ESM.docx]

**Supplementary Table 4: Metabolomics results for significant (p<0.05) changes in metabolites from week 0 to week 8 in BBP group**

| **Abbreviated** | **Super Pathway** | **p-value** | **Fold change** | **HMDB** |
| --- | --- | --- | --- | --- |
| **Metabolite Name** |  |  |  |  |
| 1-Met-His | Aminoacids Related | 0.02 | 0.77 | HMDB0000001 |
| 3-Met-His | Aminoacids Related | 0.03 | 0.44 | HMDB0000479 |
| C3 | Acylcarnitines | 0.02 | 0.89 | HMDB0000824 |
| Cer(d16:1/22:0) | Ceramides | 0.02 | 0.68 |  |
| Cer(d16:1/24:0) | Ceramides | 0.01 | 0.72 |  |
| Cer(d18:0/22:0) | Dihydroceramides | 0.05 | 0.73 | HMDB0011765 |
| Cer(d18:1/18:0) | Ceramides | 0.05 | 0.94 | HMDB0004950 |
| Cer(d18:2/24:0) | Ceramides | 0.04 | 0.96 |  |
| DG(16:1/18:2) | Diacylglycerols | 0.01 | 0.49 | HMDB0007132 |
| DG(18:2/20:4) | Diacylglycerols | 0.03 | 0.44 | HMDB0007257 |
| HArg | Aminoacids Related | 0.04 | 0.79 | HMDB0000670 |
| HexCer(d18:1/16:0) | Glycosylceramides | 0.02 | 0.90 |  |
| HipAcid | Carboxylic Acids | 0.02 | 3.65 | HMDB0000714 |
| Hypoxanthine | Nucleobases Related | 0.01 | 0.81 | HMDB0000157 |
| Ind-SO4 | Indoles Derivatives | 0.02 | 0.61 | HMDB0000682 |
| Kynurenine | Aminoacids Related | 0.03 | 0.86 | HMDB0000684 |
| Orn | Aminoacids Related | 0.00 | 0.78 | HMDB0000214 |
| p-Cresol-SO4 | Cresols | 0.03 | 0.49 | HMDB0011635 |
| PC aa C32:3 | Glycerophospholipids | 0.03 | 0.87 | HMDB0007876 |
| PC aa C38:1 | Glycerophospholipids | 0.04 | 0.59 | HMDB0007894 |
| PC aa C40:3 | Glycerophospholipids | 0.02 | 0.84 |  |
| PC aa C42:2 | Glycerophospholipids | 0.04 | 0.76 |  |
| PC aa C42:4 | Glycerophospholipids | 0.05 | 0.91 | HMDB0008191 |
| PC ae C32:2 | Glycerophospholipids | 0.03 | 0.86 | HMDB0013411 |
| PC ae C36:0 | Glycerophospholipids | 0.05 | 0.89 | HMDB0013406 |
| SM C20:2 | Sphingolipids | 0.03 | 0.84 |  |
| SM C26:1 | Sphingolipids | 0.04 | 0.53 | HMDB0013461 |
| TG(14:0/34:1) | Triacylglycerols | 0.04 | 1.43 |  |
| TG(14:0/34:3) | Triacylglycerols | 0.02 | 1.91 |  |
| TG(16:0/30:2) | Triacylglycerols | 0.01 | 2.24 |  |
| TG(16:0/32:1) | Triacylglycerols | 0.04 | 1.25 | HMDB0044726 |
| TG(16:1/32:0) | Triacylglycerols | 0.01 | 1.99 | HMDB05359 |
| TG(18:0/36:5) | Triacylglycerols | 0.02 | 0.52 | HMDB05426 |
| TG(20:2/34:2) | Triacylglycerols | 0.01 | 1.94 |  |
| TG(20:4/32:0) | Triacylglycerols | 0.03 | 1.68 | HMDB05363 |

p-values from paired t-tests. HMDB: The Human Metabolome Database. Fold changes above and below 1 indicate, respectively, an increase or a decrease in the metabolite abundance following the blueberry supplementation.
